# Comment on “Association between Lifetime Exposure to Inorganic Arsenic in Drinking Water and Coronary Heart Disease in Colorado Residents”

**DOI:** 10.1289/ehp.1509791

**Published:** 2015-07-01

**Authors:** Ragnar Rylander

**Affiliations:** Bio Fact Environmental Health Research Centre, Lerum, Sweden

In a recent issue of *Environmental Health Perspectives*, James et al. reported that life-time exposure to low levels of inorganic arsenic in drinking water was associated with increased risk of coronary heart disease (CHD). The study was well performed and reported, except for the omission of two possible confounders related to an individual’s drinking water—magnesium content and total hardness.

Several studies worldwide have demonstrated a relationship between low levels of magnesium in drinking water, or softness, and an increased risk of death from myocardial infarction (Rylander 2014). A recent study from Serbia examined populations in three different locations with varying concentrations of calcium and magnesium in their drinking water (Rasic-Milutinovic et al. 2012). The investigators observed significantly lower diastolic blood pressure as well as lower levels of serum triglycerides and creatinine in the area with the highest total water hardness. These epidemiological observations are supported by extensive studies on cell function in relation to magnesium homeostasis (de Baaij et al. 2015). From a chemical point of view, a lack of major minerals in drinking water may affect the solubility or bioavailability of arsenic. Further work to clarify the role of these agents is required before drawing final conclusions regarding the relationship between arsenic and cardiovascular disease.
